# Design and Evaluation of a Simulation-Based Program to Enhance Inter- and Intraprofessional Communication in Medical Education

**DOI:** 10.1177/23821205251414795

**Published:** 2026-03-11

**Authors:** Marielle Jambroes, Anna Kersten, Rianne Poot, Carlotta Neef, Marjo Wijnen-Meijer

**Affiliations:** 1 Dept Julius Center, Public Health, 8124 University Medical Center Utrecht, Utrecht University, Utrecht, the Netherlands; 2 Centre for Entrepreneurship, Utrecht University, Utrecht, the Netherlands; 3 Institute of Medical education, 9169 Medical Faculty and University Hospital Carl Gustav Carus, TUD Dresden University of Technology, Dresden, Germany

**Keywords:** Simulation-based education, undergraduate education, interprofessional skills

## Abstract

To enhance teamwork and patient safety, it is crucial to implement training in both inter- and intraprofessional communication within healthcare setting. Simulation-based learning provides a practical and structural approach, offering realistic, hands-on experiences to enhance communication skills. This study used a descriptive mixed-methods design to evaluate a 4-day simulation-based training programme developed to enhance the inter- and intraprofessional communication skills of medical students. A total of 216 students (eight cohorts) participated. The students participated in a variety of activities designed to stimulate the various roles of physicians, including hospital physicians, general practitioners, and public health physicians. These activities included the performance of authentic tasks such as the composition of referral letters, consultation with colleagues, and participation in multidisciplinary meetings (MDMs). The programme incorporated interactive assignments, standardized patient interactions, and real-time feedback, in addition to reflection and formative assessment, with the objective of reinforcing skills and preparing students for collaborative practice in a variety of healthcare settings. Quantitative evaluation results showed that students rated the outpatient clinic component with a mean score of 4.3 and the MDM with 3.3 on a five-point scale (1 = poor, 5 = very good). After participation in the simulation students perceived enhanced communication skills and increased understanding of the importance of collaboration across healthcare disciplines. Participants expressed a high level of satisfaction with the authenticity of the tasks and reported an increased sense of preparedness for their clerkships. These findings serve to demonstrate the perceived effectiveness of simulation-based education in the context of medical training. As the results are based on self-reported perceptions, they reflect perceived rather than demonstrated efficacy.

## Background

Rising numbers of patients with multimorbidity and the shift of hospital care to primary care are resulting in evolving healthcare needs.^1,2^ Patients frequently visit different healthcare specialists in- and outside the hospital. Recent studies continue to highlight the importance of interprofessional education (IPE) and simulation-based learning in addressing these evolving healthcare challenges. A 2025 study demonstrated that IPE interventions significantly improve collaborative competencies and communication across healthcare settings.^3^ To guarantee high-quality patient-centred care, collaboration and communication between healthcare professionals working intra- and extramural are pivotal.^4^ Therefore, future healthcare professionals should be trained in interprofessional collaborative skills.^5^ It is fundamental to recognize the importance of early and continuous IPE in promoting collaboration across healthcare professions, thereby enhancing the quality of patient care.^6^ IPE is frequently used as a mechanism to enhance the development of collaborative practice and improvement of services.^7^

Research indicates that the systematic integration of IPE throughout health professions’ education fosters better coordination of care and equips students to manage the growing complexity of healthcare systems.^6^ In accordance with this, extant literature suggests that medical students generally value IPE as a meaningful way to enhance communication skills and build mutual respect – foundational elements for patient safety and effective collaborative clinical practice.^8^

The perception among medical students is predominantly positive, with IPE being acknowledged as a valuable tool for enhancing teamwork and interpersonal skills, which are essential components for effective clinical practice.^8^

Unfortunately, IPE is predominantly used to improve collaboration between different healthcare professionals in one setting, for example in a hospital or primary care practice.^7^ However, communication and teamwork extend beyond multidisciplinary teams when patients shift from a hospital care setting to homecare and from medical specialist care to general practitioner care. Communication and collaboration between physicians across different settings (ie, intraprofessional) are as significant to practice as interprofessional communication with one setting.

As students have highlighted, the IPE has been demonstrated to assist in the reduction of misunderstandings and the improvement of mutual respect among different professional groups, which is essential for patient safety.^8^

It has been demonstrated that early exposure to clinical environments has a positive impact on students’ professional skills and their appreciation for collaborative work. Consequently, this prepares them more effectively for real-world healthcare settings.^9^

Furthermore, early clinical exposure has been shown to enhance students’ awareness of the importance of professionalism and interprofessional collaboration, thereby supporting their movement to engage in effective teamwork at the start of their education.^9^

Therefore, integrating IPE with early clinical experiences accelerates the development of inter- and intraprofessional collaborative competencies, which are pivotal for ensuring safe and effective patient care^8,9^

Taken together, these findings indicate that integrating IPE with early clinical exposure offers a comprehensive framework for equipping future healthcare professionals to respond effectively to the increasingly complex needs of healthcare systems.^6^

## Methods

### Course Design

The Medical School at Utrecht, the Netherlands, offers a 6-year curriculum, with clerkships organized into five semi-longitudinal integrated clerkships (LICs) in year 3 to 5.^7^

These LICs focus on multiple disciplines. Preceding each 12-week long integrated clerkship, students undergo a 6-week preparatory course to equip them with knowledge and skills necessary for the upcoming clerkships.

In one of the LICs Ear Nose and Throat (ENT), Ophthalmology, Dermatology, Family Medicine and Public Health collaborate. This LIC is unique as it represents all medical settings: clinical care, primary care and preventive care or public health.

Within this LIC, we designed and implemented IPE in the course period, focusing on both interprofessional and intraprofessional communication, which are among the core competencies of IPE.^5^ We used simulation-based education (SBE) as the primary instructional method.

SBE offers students the opportunity to rehearse inter- and intraprofessional communication in a safe environment and receive feedback to better prepare them for their clerkships.^10^

Based on group interviews with two groups of students (five and six students each) about their experiences with inter- and intraprofessional collaboration during the clerkships and their perceived educational need for interprofessional collaborative practice, the key learning goals for the simulation were formulated.

These goals included:

1. Students develop the ability to communicate effectively with patients, families, communities and professionals in healthcare and other fields in a responsive and responsible manner, contributing to health promotion, disease prevention and treatment.
2. Students gain experience working in different physician roles across different healthcare settings, including hospital care, primary care and public health.

In order to meet the learning goals, we developed a 4-day mandatory course for cohorts of 24-36 students (see Table 1). Each cohort starts with a 1-day training programme followed by a 3-day simulation program.

**Table 1.** Detailed Schedule of the 4-Day Simulation-Based Training Program, Including Student Role Rotations, Clinical Tasks and Reflection Activities.

Programme Day 1: - Introduction to the program. - Skills training in communication, eg, giving and receiving feedback; writing referral and discharge letters. - Training of becoming aware of prejudices about other health professions.				
Programme Days 2–4: - Students are divided into three different physician roles, either working as hospital physician, general practitioner or public health physician and change these roles each day. - Each day starts with a short briefing and role identification. - Each day ends with self- and peer-reflection by giving feedback to each other. - In the morning, students run an (online) outpatient clinic. - In the afternoon, students participate in a multidisciplinary meeting (MDM). - At the end of day, 4 students write a reflection paper regarding the learning goals.				
Timeschedule	Day 1	Day 2	Day 3	Day 4
		ENT physician General practitioner Youth health physician	Dermatologist General practitioner Infectious disease control physician	Ophthalmologist General practitioner Occupational health physician
9–9.30 am	Skills training	Role identification	Role identification	Role identification
10 am – 1 pm	Skills training	Outpatient clinic	Outpatient clinic	Outpatient clinic
1–1.30 pm	Lunch break	Lunch break	Lunch break	Lunch break
1.30–3 pm	Skills training	MDM & preparation time	MDM & preparation time	MDM & preparation time
3–4 pm	Skills training	Reflection and feedback	Reflection and feedback	Reflection and feedback

ENT, Ear Nose and Throat.

Students rotate through three different physician roles, either working as hospital physicians, family practitioner or public health physician.

### Assignments

Students first interactions with patients occur during their clerkship. To prepare them, the simulation incorporates a variety of interactive assignments.

In the outpatient clinic, every 10 min, a new patient case is presented online, totalling 15 cases. These cases are presented through an electronic learning platform (Blackboard). In the afternoon, students participated in a multidisciplinary meeting (MDM), where they discussed the cases in their assigned roles. The assignments during the outpatient clinic are designed to reflect real tasks, such as writing discharge or referral letters, consulting other physicians, and call patients to explain results or to advise schoolteachers on preventing infectious diseases. To mimic the authentic outpatient clinic, a no-show patient was included. Students learn to switch quickly between cases and to make decisions despite sometimes feeling insufficiently prepared to do so. The assignment of the MDM differs per day; for example, they need to manage an outbreak of scabies, develop a plan to prevent hearing loss in children or discuss a medical error.

### Interaction with Peers and Standardized Patients

In the outpatient clinic, a few assignments include consultations with another physician (which is a peer). Also, during the MDM students from different disciplines come together to work on a case. That is why students are urged to communicate in a professional manner, whenever interaction with a peer occurred.

Standardized patients, briefed in advance and provided with a role-play script, also participated in the simulation. Students must adapt their communication styles to interact with various audiences, learning how to adjust their style depending on whether they are speaking with a physician or a patient.

### Feedback and Reflection

Since the core learning objectives focus on communication and interprofessional collaboration, structured feedback is provided to facilitate student reflection on these competencies. Formative assessment is used to assess both interprofessional and intraprofessional skills.

On each of the assignment's, students receive online feedback on their communication skills by the physicians and standardized patients right after finishing their assignment. Physicians provide feedback on whether the request for consultation was clear, whether the consultation was complete but brief, and whether it was an efficient and effective inter-physician consultation. Patients provide feedback on whether students asked for consent to discuss, and whether they allowed room for patient questions. Both physicians and patients had room for extra comments.

During lunchtime, the answers on the patient cases became available online so students get the opportunity to check whether they treated their patients according to the standard of care.

At the end of each day, students reflect on their performance and communication skills, as well as the feedback they received from peers, physicians and standardized patients. By the end of the 4 days, students write a reflection paper, summarizing their key learning experiences, their key takeaways and how they plan to apply their learning during their upcoming clerkships.

### Course Evaluation

As part of the routine curriculum evaluation using locally developed evaluation forms, students completed course evaluations. The form consisted of eight topics, one of which focused on educational concepts. Of the 36 questions related to educational concepts, three specifically referred to the simulation course: ‘*The training day sufficiently prepared me for the simulation’*, ‘*The outpatient clinic was a valuable learning experience’*, and ‘*The MDM was a valuable learning experience’*.

In addition, we extended routine closing sessions at the end of the first three courses, in which students were invited to reflect on their experiences with SBE with a specific focus on communication skills and interdisciplinary collaboration. Participation was voluntary, snacks were provided and almost all students participated in these sessions. During these sessions, oral feedback was gathered and detailed notes were taken by the facilitators.

### Ethics Statement

According to Dutch law, no formal ethical approval was required for this study as the evaluation was part of routine evaluation embedded in the curriculum.

### Analysis

Quantitative data from the evaluation forms were analysed descriptively. Notes from the extended evaluation sessions were analysed thematically by MJ. Initial open coding was followed by comparison across responses. Thematic tables were used to structure the data and to explore both shared and divergent student experiences.

## Results

From September 2017 to September 2018, eight cohorts comprising a total of 216 students participated in the simulation program. In total 106 students completed an evaluation form. Overall evaluation scores from the students for the outpatient clinic were 4.3 (SD 0.9) and for the MDM, the score was 3.3 (SD 1.1) on a 5-point scale (1 = poor, 5 = very good).

The analysis of three feedback sessions, each involving approximately 20 students, indicated that students generally appreciated the educational concept, describing it as both novel and engaging. Across cohorts, there was substantial consensus regarding their experiences, which could be organized into three themes: 1) learning through applying prior knowledge, 2) experiencing realism and role enactment and 3) learning through feedback and reflection.

Day one of the simulation weeks was frequently characterized as disorienting. Students expressed a need for a clearer logistical introduction, ideally supplemented with concrete examples and predefined expectations, to help them navigate the learning activities more effectively.

### Learning Through Applying Prior Knowledge

Students reported that the simulation exercises were most beneficial when they could draw on recent curricular content. When cases aligned with specialties that had been covered in the preparatory course (eg, dermatology or ENT), students described more confident engagement and deeper learning. In contrast, when knowledge was lacking, particularly in public health, students defaulted to copy-pasting from guidelines without understanding.

*With dermatology I could really think through the case. But as a preventive youth health doctor, I was just copying from the standard — I didn’t really learn much.*

### Experiencing Realism and Role Enactment

Despite knowing it was a simulation, students described moments where they ‘felt like a real doctor’, particularly during telephone consultations. Time pressure and responsibility for decision-making contributed to a sense of urgency and authenticity.

*Once I pick up the phone, I’m completely in the role. It feels real — like during a clinical rotation.*

Students valued opportunities to independently manage cases from intake to communication and collaboration with different disciplines. The role as public health physician was most difficult to engage with as prior knowledge was lacking. They perceived the simulations as more engaging and reflective of actual clinical tasks than traditional assignments.

### Learning Through Feedback and Reflection

Feedback emerged as a central learning mechanism. Especially after phone consultations, students reported being surprised by the discrepancy between what they thought they had said and how it was perceived by the receiver. These moments encouraged reflection and adjustment.

*I was sure I had explained everything, but the feedback said otherwise — that really made me think.*

Students appreciated the safety of the environment, noting they were more willing to try, fail and learn than in clinical settings.

## Discussion

Students highly valued the safe environment in which they could practice their skills. They particularly appreciated the authenticity of the communication tasks and the just-in-time feedback provided. The fast-paced nature of the outpatient clinic, where new patient cases emerged in quick succession, was perceived as beneficial because it taught them to manage time effectively and make decisions under pressure. Moreover, practicing communication with doctors from different disciplines under such conditions was regarded as essential preparation for real-life clinical work. At the same time, students emphasized that having sufficient disciplinary knowledge was crucial to fully engage with and appropriately enact their professional role.

Overall, the feedback indicated that students not only felt better prepared for their upcoming clerkships in terms of communication but also developed stronger awareness of their professional role within the team.

Recent findings support the idea that IPE fosters professional awareness and identity. A recent review found IPE simulation experiences help students understand their roles in healthcare teams and recognize the value of their profession.^11^

However, the main aim of this article was to describe how we implemented SBE as a method for training interprofessional competencies. We do present results regarding students’ perceptions of the simulation, corresponding to the first level of the Kirkpatrick model, but the implementation was not designed as an evaluation study. This constitutes an important limitation of our findings. One potential explanation for the lower evaluation scores of the MDMs in comparison to the outpatient clinic may be the more abstract nature of the MDM assignments. While the outpatient clinic cases centred on individual patient encounters with clear clinical decisions, the MDMs addressed broader public health and systemic issues. It is possible that students at this stage of their training may have found these issues to be less tangible or relevant, or – as they noted in the interviews – felt insufficiently prepared due to their limited exposure to public health in the curriculum.

Furthermore, the absence of a direct patient narrative in the MDM cases may have rendered it more challenging for students to emotionally engage or fully comprehend the practical implications of the decisions under discussions. This could have resulted in a lower sense of perceived authenticity or impact.

These findings emphasize the significance of designing multidisciplinary tasks that are both authentic and appropriately challenging for students’ development level, potentially by anchoring broader topics in concrete patient scenarios or by adding clearer expected learning outcomes.

Many students reflected on the experience, reporting that the simulation programme had helped them understand the importance of being aware of one's own role in both interprofessional and intraprofessional collaboration. They also noted that when disciplinary knowledge was insufficient, it became more difficult to provide high-quality patient care, including effective collaboration across professions and disciplines. They noted that skills learned during the simulation were transferable to their clerkships, where they had to engage with a wide range of professionals in diverse healthcare settings. Furthermore, students highlighted how the simulation helped them develop an appreciation for the roles of various professionals in patient care, which is vital for providing high-quality, patient-centred care.

Instructors also noted the success of the program in enhancing communication skills among students. The structured feedback and reflection process encouraged students to think critically about their performance and continue to improve. Students’ communication with both peers and patients was seen as more efficient and empathetic because of the simulation.

The findings of this study are in alignment with the extant literature in the field, which demonstrates that interprofessional simulation fosters collaborative behaviours and communication competence, which in turn contributes to an enhancement in attitude.^11^

Future iterations of the programme could enhance learning further by incorporating objective assessments of communication performance, such as structured observation checklists or OSCE-style evaluations, and by extending participation to students from other healthcare professions. Strengthening the preparatory content for public health-related MDMs could also help students engage more meaningfully with system-level discussions. Longitudinal follow-up studies could investigate whether perceived improvements in communication lead to observable behavioural changes during clinical placements.

Key factors contributing to the success of the program included the focus on authenticity, practicing different professional roles, the interactive learning approach and the regular feedback loops that encouraged continuous improvement. Students reported that the feedback from both physicians and standardized patients was particularly valuable. By engaging with standardized patients, students had the opportunity to practice clear and empathetic communication in a safe environment, which built their confidence and allowed them to refine their skills.

These findings align with previous research suggesting that simulation-based IPE improves students’ preparedness for collaborative practice and patient-centred care^7^ and that early clinical exposure has a positive impact on students’ recognition of the requirement for professionalism and collaborative skills.^9^

## Conclusion

The simulation-based programme is an effective method for teaching interprofessional and intraprofessional communication skills. Students mentioned increased confidence and readiness to engage in both forms of collaboration during their upcoming clerkships. As patient safety and communication skills continue to be critical priorities in medical education, simulation programmes like this one play an essential role in preparing future healthcare professionals to collaborate effectively across disciplines and settings. The success of this programme highlights the potential of using simulation to enhance communication and collaboration skills, ensuring that students are equipped to navigate the complexities of modern healthcare systems.
